# Activation of lncRNA DANCR by H3K27 acetylation regulates proliferation of colorectal cancer cells

**DOI:** 10.1007/s12672-024-01124-8

**Published:** 2024-06-28

**Authors:** Yue Han, Ti-Dong Shan, Hai-Tao Huang, Ming-Quan Song, Li Chen, Qian Li

**Affiliations:** 1 Department of Gastroenterology, The Affiliated Hospital of Qingdao University, Qingdao University, 16 Jiang Su Road, Qingdao, 266000 Shandong People’s Republic of China; 2 The International Medical Department, the Affiliated Hospital of Qingdao University, Qingdao, Shandong 262000 People’s Republic of China

**Keywords:** DANCR, miR-508-5p, Colorectal cancer, Proliferation, H3K27 acetylation

## Abstract

The long noncoding DANCR functions as a tumor oncogene in many cancers, including colorectal cancer (CRC). However, the molecular mechanism of DANCR in CRC has not been explored. This study probed the function and potential mechanism by which DANCR contributes to the progression of CRC. The obtained data indicated that DANCR is overexpressed in CRC tissues and cell lines. Knockdown of DANCR hindered CRC cell proliferation, which was mediated by cyclin D1 and CDK4. Bioinformatic analysis, luciferase reporter assays and subcellular fractionation verified that DANCR directly binds to miR-508-5p. Moreover, DANCR acts as a miR-508-5p ceRNA to regulate expression of ATF1. In addition, upregulation of DANCR is attributed to H3K27 acetylation at the promoter region. In conclusion, our study confirmed that activation of lncRNA DANCR by H3K27 acetylation has an oncogenic role in CRC progression and provides a potential therapeutic target for CRC.

**Supplementary Information:**

The online version contains supplementary material available at 10.1007/s12672-024-01124-8.

## Introduction

Colorectal cancer (CRC) is the third most common tumor in the world and also the fourth most common cause of death [1]. CRC is the result of the combined effect of heredity and the environment [2]. Although much experience has accumulated in the treatment of CRC, survival based on drug resistance and metastasis is still a challenge [3]. To cope with these difficult clinical problems, it is particularly important to find effective and safe new molecules to diagnose and treat CRC.

Studies have shown that nonencoded RNA can be used as an important marker for the potential diagnosis, treatment and prognosis of tumors [4]. Long noncoding RNAs (lncRNAs) are nonprotein-coding RNAs that are more than 200 nucleotides in length and function by interacting with genes and proteins. As reported, lncRNAs participate in various cellular processes in cancer cells [5]. Indeed, lncRNAs perform numerous biological functions, such as proliferation, invasion and autophagy [6]. MicroRNAs (miRNAs) are 18- to 25-nucleotide noncoding RNAs that modulate expression of mRNAs [7]. LncRNAs compete with miRNAs to reduce the binding of miRNAs and mRNAs, which further affects expression of target genes [8].

It was recently found that differentiation antagonizing non-protein coding RNA (DANCR) is aberrantly expressed in different tumors, including CRC [9–11]. It has also been reported that roles of DANCR in the carcinogenesis, with an especial emphasis on its role in the development of osteosarcoma and lung, liver, pancreatic and CRC [11]. Study also showed DANCR may potentially provide a promising future therapeutic strategy for breast cancer treatment [12]. Previous research has reported that DANCR played a critical role in Doxorubicin-induced apoptosis in colorectal cancer cells through regulating the expression of MALAT1 [13]. Overall, although DANCR has been featured in several studies of CRC, the specific mechanism of DANCR still needs to be further studied. Moreover, to the best of our knowledge, there has been no research to date reporting the mechanism underlying the relationship between DANCR and ATF1 in CRC. In this study, DANCR was assessed in CRC tissues and cell lines. Moreover, the effects of DANCR on malignant behavior were detected through functional experiments, and the potential mechanisms of DANCR in CRC are herein discussed in depth. This study may provide new directions for therapeutic strategies for CRC.

## Materials and methods

### Patients and tissue samples of CRC

Sixty-nine fresh frozen CRC tissue and adjacent nontumor tissue samples (sampled at more than 5 cm from the tumor) were collected from The Affiliated Hospital of Qingdao University and stored at − 80 °C for this study. All patients signed the informed consent form for research purposes. The Hospital Ethics Review Committee approved the procedures of this study.

### Cell lines and culture conditions

Human normal cell lines (NCM460) and human CRC cell lines (SW480, DLD-1, RKO, LOVO and HCT-116) were purchased from the Institute of Biochemistry and Cell Biology of the Chinese Academy of Sciences (Shanghai, China). The cells were cultured as follows: in Roswell Park Memorial Institute (RPMI; Gibco, Grand Island, NY, USA) 1640 containing 10% FBS with 5% CO2 at 37 °C. C646 (Selleck Chemicals, Houston, TX, USA) was used at 10 μmol/L for 48 h, as needed.

### RNA extraction and real-time quantitative polymerase chain reaction (qPCR)

Total RNA from CRC tissues and cell lines was extracted using TRIzol reagent (Invitrogen, Grand Island, NY, USA); mRNA was reverse transcribed to cDNA using PrimeScript RT Reagent Kit (Takara, Dalian, China). The DANCR expression level was measured using a SYBR Green PCR Master mix kit (Takara, Dalian, China). The sequences of the DANCR primers used were as follows: forward, AGT TCT GAC CAC GAG CTT TTC; reverse, GGT GCT ATG AGA TTC CGA GTT C. GAPDH was employed as an endogenous control. The 2^−ΔΔC^t method was used to calculate the results. The other primers used are listed in Table S1.

### Cell transfection

The two si-DANCR molecules (si#1 and si#2), miR-508-5p mimics and inhibitors were obtained from GenePharma (Shanghai, China). Cells were cultured to 70% confluence. Lipofectamine 3000 Transfection Reagent (Life Technologies, Grand Island, NY, USA) was applied for cell transfection according to the manufacturer’s protocol. The sequences of the primers used are shown in Table S1.

### Dual-luciferase reporter plasmid transfection

RB-REPORT™ plasmids (DANCR-wnt and ATF1-wnt) and their mutation plasmids (DANCR-mut and ATF1-mut) were obtained from RiboBio (Guangzhou, China). A Dual-Luciferase Reporter Assay system (Promega, Madison, WI, USA) was used to detect firefly luciferase and Renilla luciferase, and the activity of the former was normalized to that of the latter.

### Cell counting kit-8 (CCK8) assay

A cell counting kit (CCK8 assay, Dojindo, Kumamoto, Japan) was used to measure the proliferation of CRC cells. A total of 2000 CRC cells were transfected with siRNAs and plated into 96-well plates for 24, 48, 72, and 96 h. A multifunctional microplate reader SpectraMax M5 (Sunnyvale, CA, USA) was used to measure absorbance at 490 nm.

### In situ hybridization

DIG-labeled LNA-DANCR was designed and synthesized by RiboBio (Guangzhou, China). In brief, paraffin-embedded tissues were washed and permeabilized with Triton X-100 solution and then hybridized at 37 °C overnight. Diaminobenzidine solution (1:900; Boster Biological Technology) was used to determine expression. A BX51 microscope (Olympus Corporation) was used to observe staining intensity at a magnification of × 400.

### Subcellular fractionation

A Nuclear and Cytoplasmic Protein Extraction Kit (Beyotime, Shanghai, China) was used to extract and detect cytoplasmic and nuclear RNA. DANCR expression levels in the cytoplasmic and nuclear fractions were measured by qPCR. GAPDH and U6 were used as cytoplasmic and nuclear controls, respectively.

### Chromatin immunoprecipitation assay (ChIP)

An EZ ChIP™ Chromatin Immunoprecipitation Kit (Millipore, Bedford, MA, USA) was used to conduct ChIP. Briefly, tissues and cells were incubated with formaldehyde, and DNA‒protein crosslinks were generated for 20–30 min. Then, the crosslinked chromatin was sonicated into fragments. Both anti-H3K27ac (1:1,000; Abcam, Cambridge, UK) and anti-IgG (negative control) antibodies were used for immunoprecipitation. The precipitated DNA was analyzed using qPCR.

### Protein extraction and western blotting

Total proteins were extracted from CRC tissues and cell lines with RIPA buffer (Thermo Fisher Scientific, Waltham, MA, USA). The protein lysates were separated by 10% SDS‒PAGE and transferred to PVDF membranes (Sigma), which were blocked with 5% skim milk for 2 h at room temperature. The membranes were incubated with primary antibodies (ATF-1 antibody (#25177; 1:1000) and β-actin antibody (#8457; 1:1000); Cell Signaling Technology, Beverly, MA, USA) overnight at 4 °C. Then, the membrane was incubated with HRP-labeled secondary antibody (Cell Signaling Technology, USA) for 1 h at room temperature. The protein bands were detected by an enhanced chemiluminescence kit (Millipore, Billerica, Massachusetts, USA).

### Statistical analysis

Results are expressed as the mean ± standard deviation (SD) from six separate experiments. Comparisons between groups were analyzed using Student's test, and multiple group comparisons were analyzed using one‑way ANOVA with Tukey's post hoc test. Correlations between lncRNA and CRC clinical characteristics were determined using Pearson's chi‑squared test. All statistical analyses were performed using SPSS 22.0 (IBM Corp.). *P* < 0.05 was indicative of a significant difference.

## Results

### DANCR was overexpressed in CRC tissues and cells

**Fig. 1:**
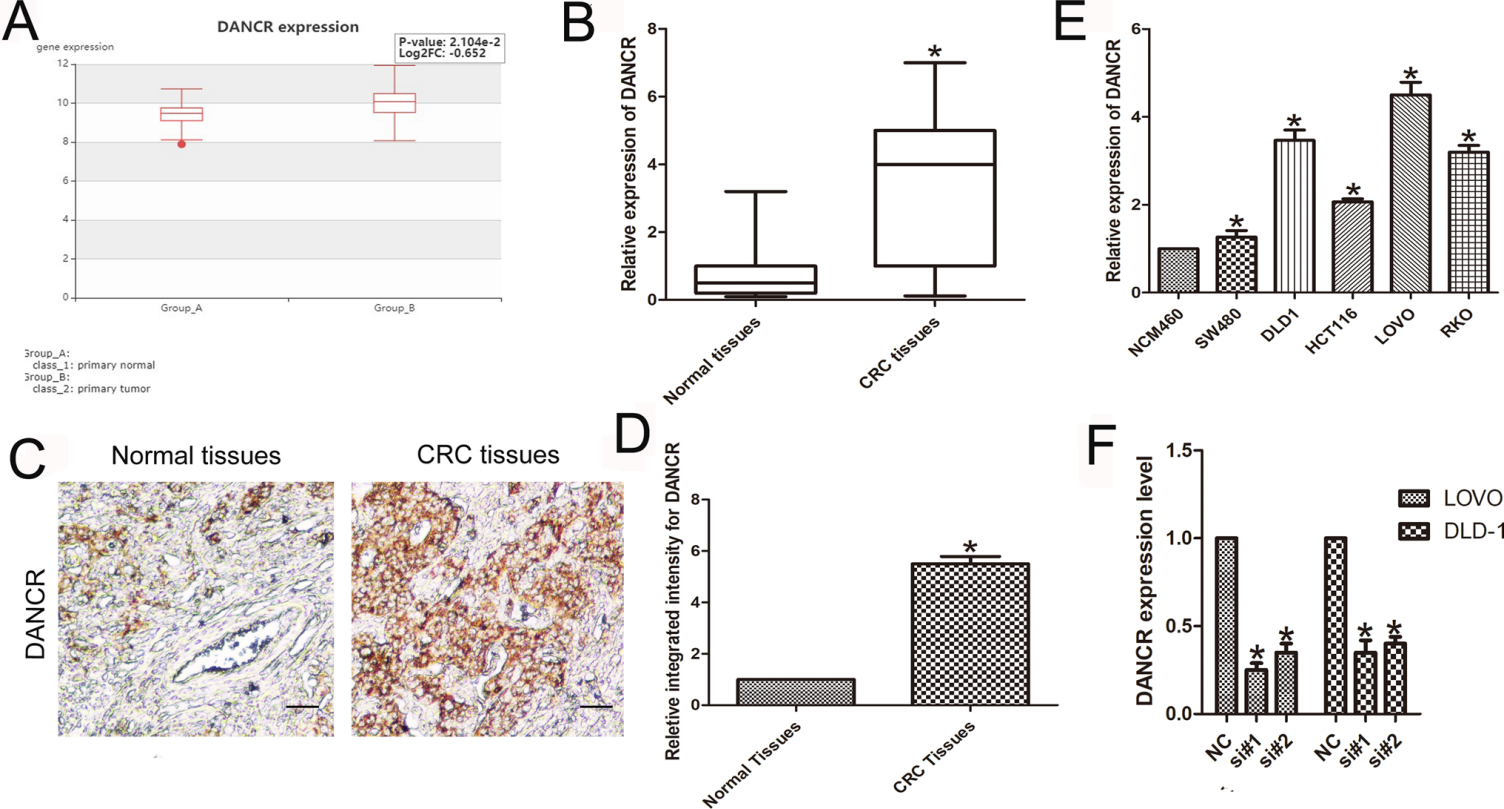
Abnormal DANCR expression in CRC and cell lines. **A** The HCMDB dataset showed the expression level of DANCR in CRC tissue (*P < 0.05). **B** Expression of DANCR was significantly elevated in CRC tissues. Statistical differences were analyzed using the Wilcoxon signed-rank test (*P < 0.01). **C**, **D** In situ hybridization of a DIG-labeled LNA- DANCR probe showed that DANCR was mainly distributed in the cytoplasm of CRC tissue. **E** Expression of DANCR in CRC cell lines (LOVO, HCT-116, DLD-1, SW480, and RKO) was detected by qPCR. **F** Knockdown efficiencies in cells transfected with si-DANCR. (n = 6; *P < 0.05 vs. NC)

Based on the HCMDB dataset (http://hcmdb.i-sanger.com), we identified that DANCR was overexpressed in CRC tissue compared with matched adjacent normal tissue (n = 6; *P* < 0.05; Fig. 1A). Data from CRC tissue specimens also showed that the expression level of DANCR was higher in CRC tissue than in normal tissue (n = 6; P < 0.01; Fig. 1B). Additionally, using in situ hybridization with a DIG-labeled LNA-DANCR probe, we discovered that DANCR was predominantly located in the cytoplasm of CRC tissue and that its expression was higher than that in normal tissue (n = 6; *P* < 0.05; Fig. 1C and D). Furthermore, expression of DANCR, as measured by qPCR, was upregulated in CRC cell lines (SW480, DLD-1, RKO, LOVO and HCT-116; n = 6; *P* < 0.01; Fig. 1E). Among these CRC cells, LOVO and DLD-1 cells were much more abundant for the following study. To further identify the roles of DANCR in CRC cell behaviors, DANCR expression in both cell lines was silenced with two siRNAs (si#1 and si#2). Compared with that in the control group, the silencing efficiencies were significantly reduced (n = 6, *P* < 0.05; Fig. 1F). These results showed high expression of DANCR in CRC cells.

### Silencing of DANCR reduced proliferation in CRC cells

**Fig. 2:**
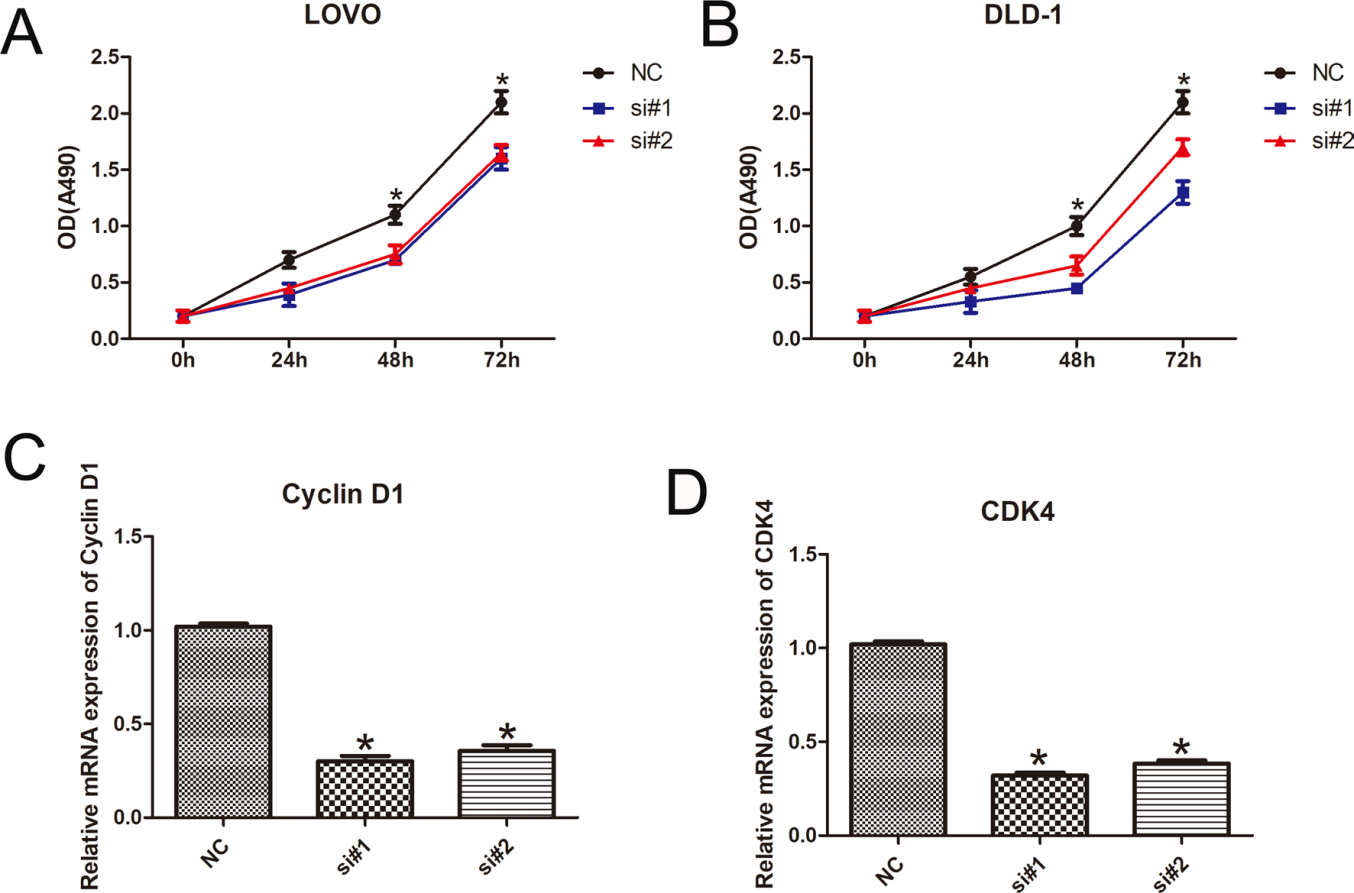
DANCR knockdown inhibits proliferation of CRC cells. **A**, **B** CCK-8 assays revealed that knockdown of DANCR inhibited proliferation in cells. **B** Histological analysis of rates of colony formation in the control (NC) and DANCR knockdown groups. **C**, **D** qPCR showed the mRNA expression level of cell cycle-related molecules [cyclin D1 and cyclin-dependent kinase 4 (CDK4)]. (n = 6; *P < 0.05 vs. NC)

To investigate the effect of cell proliferation on the loss of DANCR function, we transfected both LOVO and DLD-1 cells with siRNAs. We found that the number of cells was significantly decreased compared with that in the control group, as evaluated by the CCK-8 assay (n = 6, *P* < 0.05; Fig. 2A and B). Meanwhile, several cell proliferation-related molecules [cyclin D1 and cyclin-dependent kinase 4 (CDK4)] were detected by qPCR assay, and the levels of cyclin D1 and CDK4 were downregulated after DANCR knockdown compared with the control group (n = 6, *P* < 0.05; Fig. 2C and D).

### DANCR sponges miR-508-5p, and its function was inhibited by miR-508-5p in CRC cells

**Fig. 3:**
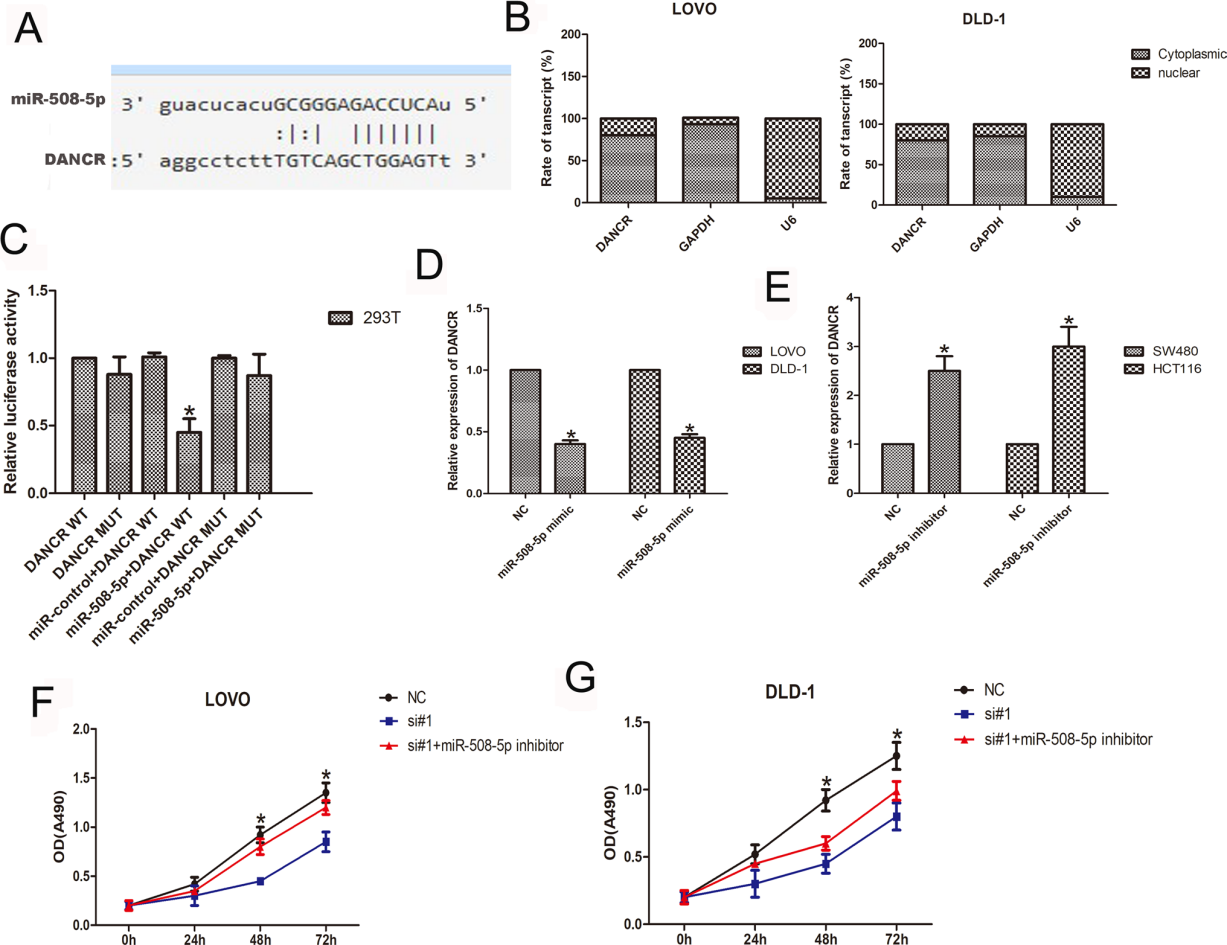
DANCR sponges miR-508-5p, and its function is suppressed by miR-508-5p. **A** Predicted miR-508-5p binding sites in the DANCR sequence. **B** Subcellular fractionation showed that DANCR was principally located in the cytoplasm of CRC cells. **C** The luciferase reporter assay showed that the luciferase activity of cells cotransfected with miR-508-5p and DANCR-WT reporter plasmids was decreased compared with that of the reporter with DANCR-mut or cotransfected miR-508-5p. **D**, **E** Cells were transfected with a miR-508-5p inhibitor or mimic, and DANCR expression was analyzed by qPCR. **F**, **G** CCK8 showed that the decreased number of live cells was partly upregulated in DANCR knockdown CRC cells. (n = 6; *P < 0.05 vs. NC; #*P* < 0.05 vs. si#1)

A bioinformatics tool (https://starbase.sysu.edu.cn/ceRNA) was used to predict any downstream regulatory correlation for miRNA/DANCR. The results highlighted that miR-508-5p potentially binds to DANCR (Fig. 3A). Moreover, in the subcellular fractionation assay, DANCR was principally located in the cytoplasm of CRC cells (Fig. 3B). A luciferase reporter assay showed that the activity of DANCR-WT but not the DANCR-mut reporter construct was inhibited by miR-508-5p mimic overexpression (n = 6, *P* < 0.05; Fig. 3C). However, we noticed that upregulation of miR-508-5p expression reduced DANCR levels but that downregulation of miR-508-5p increased DANCR expression (n = 6, *P* > 0.05; Fig. 3D and E). These results indicated that miR-508-5p binds to DANCR. Furthermore, the roles of miR-508-5p in DANCR function in CRC cell proliferation were also detected by CCK8 assays. The reduced cell proliferation of CRC cells caused by DANCR knockdown was partly overcome by miR-508-5p inhibitor overexpression (n = 6, P < 0.05; Fig. 3F and G). These data strongly indicate that DANCR is partly mediated by miR-508-5p.

### ATF1 was a target of miR-508-5p

**Fig. 4:**
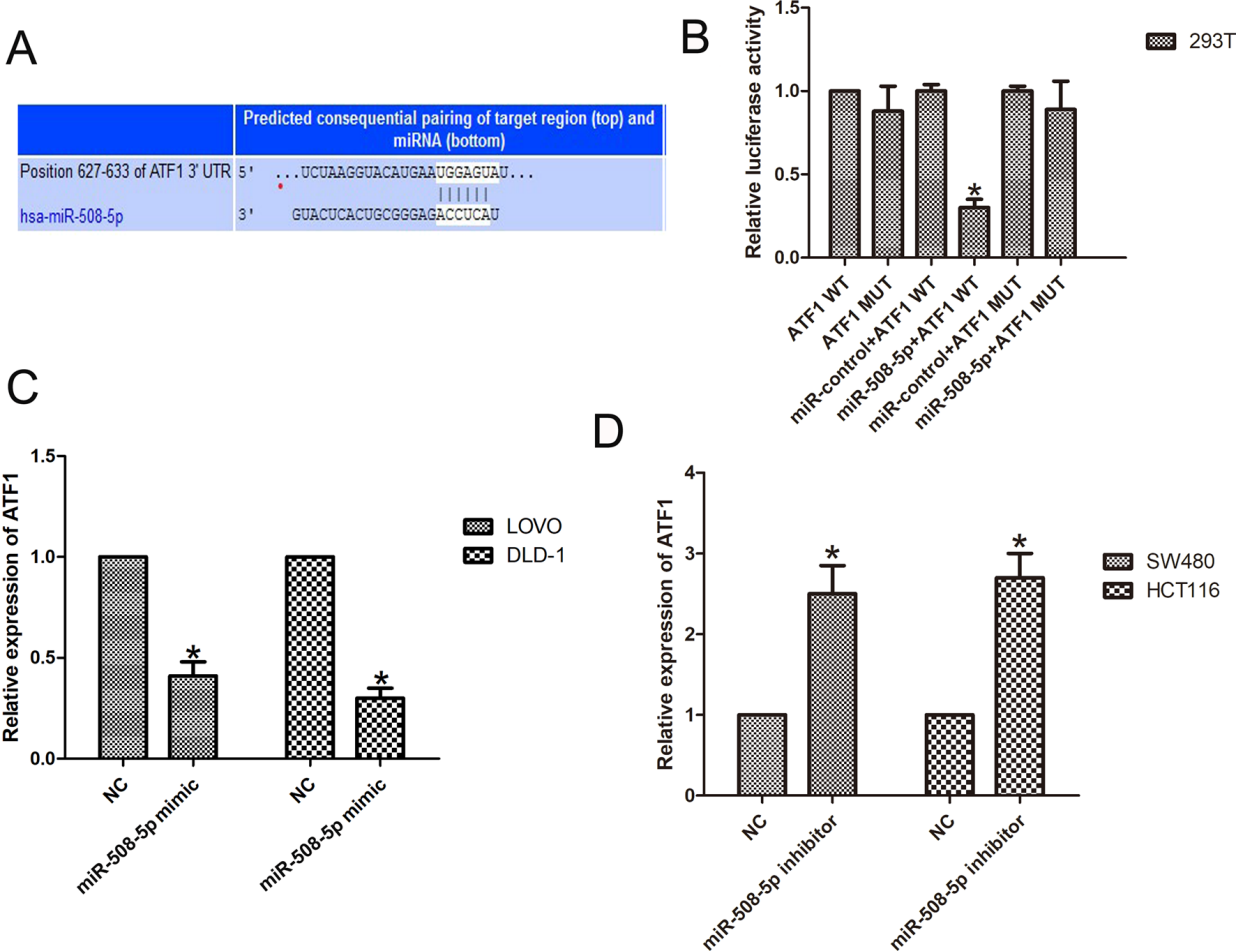
ATF1 is a target of miR-508-5p. **A** Bioinformatics tools found that ATF1 was a target of miR-508-5p. **B** The luciferase reporter system showed that after cotransfection of miR-508-5p mimics and ATF1-WT, luciferase activity was significantly reduced. **C**, **D** The results of by qPCR assays showed that the miR-508-5p mimic repressed expression of ATF1 in cells. (n = 6, *P < 0.05 vs. NC)

To explore the molecular mechanism by which miR-508-5p promotes CRC progression, we used bioinformatics tools (http://www.targetscan.org/vert_72/) to predict the potential targets directly interacting with miR-508-5p. The data showed that ATF1 directly targets miR-508-5p (Fig. 4A). Through the luciferase reporter system, we observed that cotransfection of miR-508-5p mimics and ATF1-WT dramatically reduced luciferase activity but that it did not change when ATF1-MUT was transfected into cells (n = 6, *P* < 0.05; Fig. 4B). In addition, qPCR analysis showed that ATF1 was overexpressed by miR-508-5p inhibitor transfection but was decreased by transfection with the miR-508-5p mimic (n = 6, *P* < 0.05; Fig. 4C and D).

### DANCR regulated ATF1 expression via miR-508-5p and proliferation in CRC cells

**Fig. 5:**
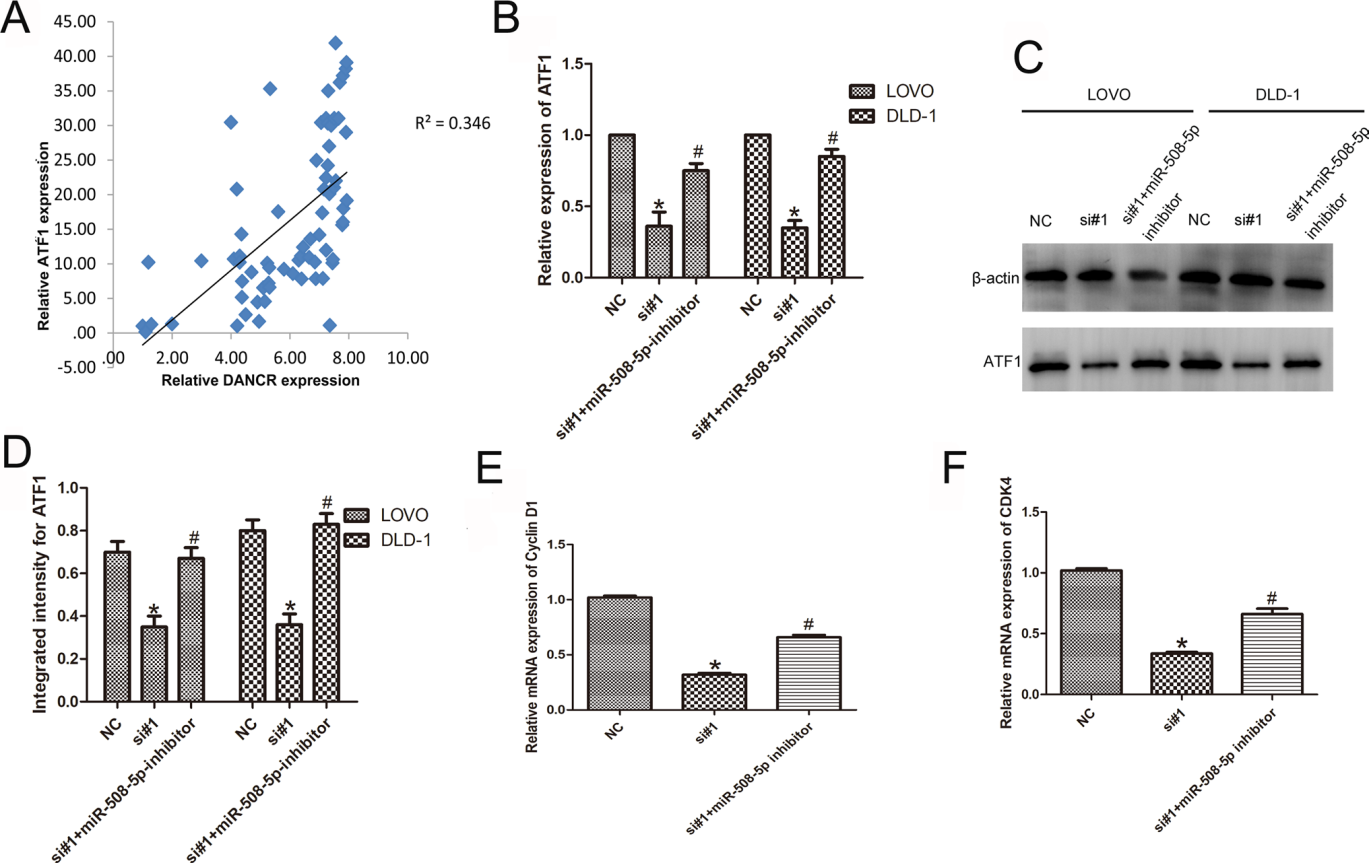
DANCR regulates ATF1 expression through miR-508-5p. **A** There was a significant positive correlation between DANCR and ATF1 (*P* < 0.01, R^2^ = 0.346). **B** qPCR results showed that knockdown of DANCR reduced expression of ATF1 but that the miR-508-5p inhibitor partly abolished this silencing effect. **C**, **D** Western blotting assays showed that ATF1 protein downregulation in DANCR knockdown cells was rescued by miR-508-5p inhibitor transfection. **E**, **F** qPCR analysis showed that the decreased levels of cyclin D1 and CDK4 in DANCR knockdown cells were partly rescued by miR-508-5p inhibitor transfection. (n = 6; *P < 0.05 vs. NC; #*P* < 0.05 vs. si#1)

qPCR showed that while DANCR was upregulated in CRC tissues, ATF1 was also upregulated in the same tumor specimens, resulting in a significant positive correlation between DANCR and ATF1 [*P* < 0.01, R^2^ = 0.346; Fig. 5A]. The qPCR results showed that knockdown of DANCR downregulated expression of ATF1, which was partly rescued by the miR-508-5p inhibitor in CRC cells (n = 6, *P* < 0.05; Fig. 5B). Consistently, Western blotting showed that DANCR knockdown notably decreased ATF1 expression but that the miR-508-5p inhibitor partly rescued ATF1 expression in cells (n = 6, *P* < 0.05; Fig. 5C and D). Next, we analyzed the abnormal proliferation behavior caused by qPCR to determine whether DANCR is caused by the miR-508-5P target ATF1. After miR-508-5p inhibitor transfection, the decreased levels of CDK4 and cyclin D1 in DANCR-knockdown cells were partially recovered (n = 6, *P* < 0.05; Fig. 5E and F).

### H3K27 acetylation activated DANCR expression

**Fig. 6:**
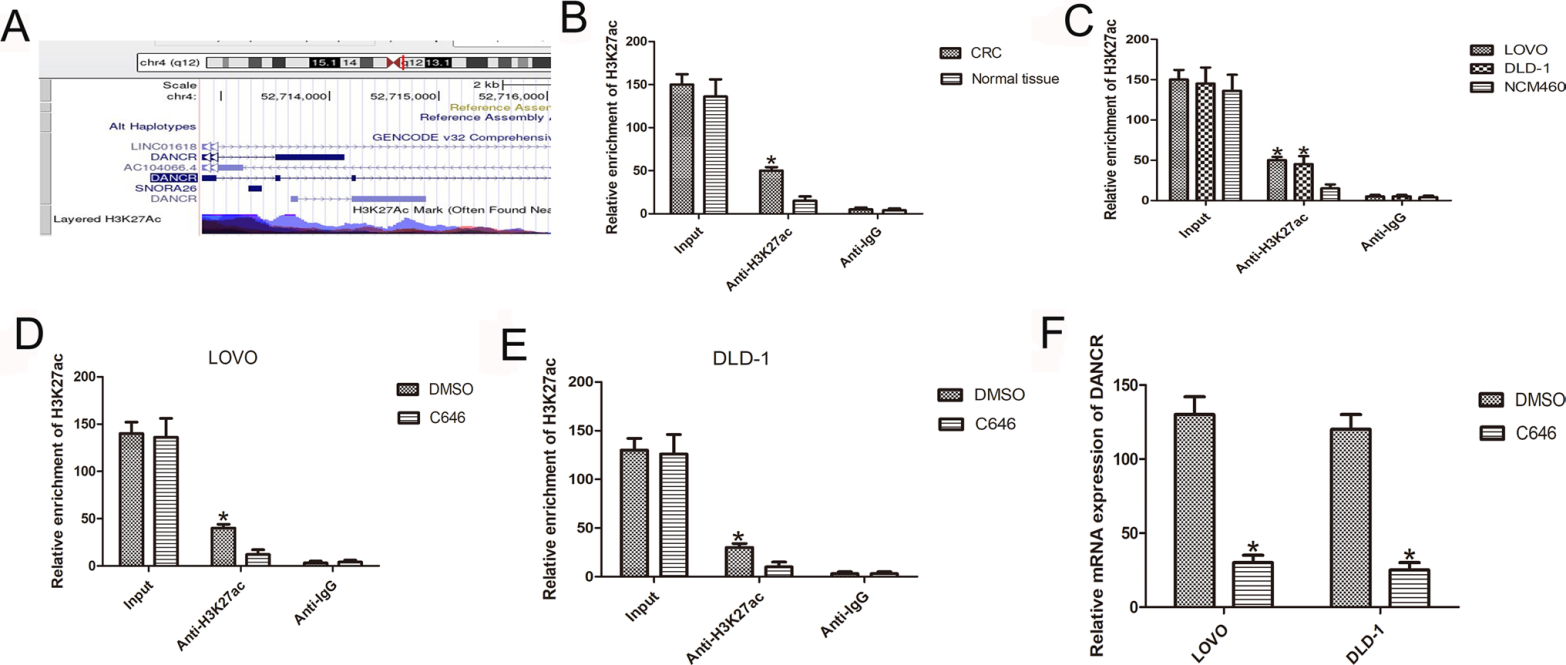
DANCR is activated by H3K27 acetylation at the promoter region. **A** Bioinformatics analysis showing high enrichment of H3K27ac at the DANCR promoter. **B** ChIP assays detecting H3K27 acetylation at the DANCR promoter in CRC tissues. **C** ChIP assays detecting H3K27 acetylation at the DANCR promoter in CRC cells. **D**, **E** ChIP assays detecting H3K27 acetylation at the DANCR promoter after treatment of CRC cells with C646. **F** DANCR expression in C646-treated CRC cells using qPCR. (n = 6, *P < 0.05 vs. NC)

The underlying mechanism of high DANCR levels in CRC cells needs to be investigated. Bioinformatics analysis (http://genome.ucsc.edu/) showed H3K27ac to be highly enriched in the DANCR promoter (Fig. 6A). To explore this, we used the anti-H3K27ac antibody of the ChIP assay in both CRC tissues and cancer cell lines. When compared with adjacent normal tissues, the expression level of H3K27ac at the DANCR promoter in CRC tissues was highly increased (n = 6, *P* < 0.05; Fig. 6B). Moreover, in CRC cells, levels of H3K27ac were enriched at the DANCR promoter (n = 6, *P* < 0.05; Fig. 6C). To assess whether H3K27ac is specific for DANCR transcription activation and expression upregulation, CRC cells were treated with C646 (a histone acetyltransferase inhibitor). Moreover, when compared with control cells, enrichment of H3K27ac was lower in CRC cells after C646 treatment (*P* < 0.05, Fig. 6D and E). We further found decreased expression of DANCR in C646-treated CRC cells (n = 6, *P* < 0.05; Fig. 6F).

## Discussion

Recent evidence has shown that lncRNAs have the ability to regulate complex and diverse biological functions, and they have important significance for different tumor types and for diagnosis, treatment and prognosis [14, 15]. In the last few years, multiple studies have established DANCR roles in promoting cancer tumor growth and migration among different cancers [16, 17]. DANCR is able to regulate hepatic malignant tumor progression via miR-125b-5p [18]. DANCR affects the resistance of gastric cancer by adjusting expression of MDR1 and MRP1 [19]. Studies have also shown the roles of DANCR in colorectal cancer tissues [20]. However, the specific mechanism through which DANCR exerts its biological functions has not yet been fully clarified. In particular, there are few studies on what causes the increase in DANCR in CRC. In this study, we found that DANCR was overexpressed, which is consistent with previous research [20]. We further found through CCK-8 experiments that DANCR knockdown can significantly inhibit the malignant proliferation of CRC cells. Studies have confirmed that CDK4 acts on cyclin D1 to further regulate cell proliferation [21–23]. The present study revealed the mechanisms of proliferation by which DANCR knockdown can reduce levels of cyclin D1 and CDK4 in cell lines. Although it would be helpful to examine the association of this gene with survival in colorectal cancer patients, samples from at least more than five years would be needed, and the samples used in this study were only obtained for at most one year. We will further evaluate DANCR and publish the survival association of this gene with colorectal cancer patients in the future.

Studies have shown that ceRNAs play an important role as lncRNAs that cause the development and progression of tumors [24]. However, in the cancer environment, in addition to lncRNA‒ceRNA signaling, many other factors might affect the occurrence and development of tumors. These factors might enhance the development of tumors or reduce the development of tumors. Such inconsistency requires further research. In this study, lncRNA‒ceRNA signaling was the focus of our research, and we found that DANCR was mainly expressed in the cytoplasm of CRC through in situ hybridization and subcellular fractionation assays. These results indicate that the ceRNA mechanism may exist and play an important regulatory role. DANCR, as a lncRNA, has many targets or targets, which might regulate CRC in different ways. Previous researches have reported that DANCR, as biomarkers for the diagnosis of colorectal cancer, might facilitate the growth and metastasis of CRC by regulating the epithelial-mesenchymal transition (EMT) process [14, 24]. Consistently, our results showed that miR-508-5p targets DANCR directly through bioinformatics analysis and dual-luciferase analysis. The results implied that DANCR acts as a sponge. Research has found that miR-508-5p plays a tumor-suppressor role in different cancers, including CRC [25, 26]. Moreover, our data showed that the effect of DANCR was partly reversed by miR-508-5p in CRC cells. Nevertheless, the patient’s genetic profile, primary tumor location and expression of DANCR could be insightful for future clinical practice in CRC therapy. This needs to be further investigated.

LncRNAs regulate the biological characteristics of cancer cells by competing with miRNAs for their shared strain [27]. In this study, we originally detected that miR-508-5p directly targets activating transcription factor 1 (ATF1). ATF1 participates in cell viability and cell transformation by adjusting the transcription of extracellular signals [28–31]. ATF1 plays a critical role in the proliferation and invasion of many cancer cells [32–34]. In this study, we found that ATF1 correlated positively with DANCR. Moreover, we validated that DANCR sponges miR-508-5p to regulate proliferation via ATF1. We also showed that a miR-508-5p inhibitor can partially inhibit the decrease in cycle-related molecules caused by DANCR knockdown. These studies have demonstrated that DANCR depletion-induced proliferation inhibition is partly mediated through ATF1 in CRC cells. According to bioinformatics website analysis (targetscan.com), whether in vivo or in vitro, a miRNA can have many target genes, and one target gene can correspond to multiple miRNAs. The series of in vitro experiments helps to verify our hypothesis. The in vivo experiments indicate relationships between miRNAs and target genes, at least partially. This needs to be further investigated.

Overall, the underlying mechanism causing DANCR overexpression in CRC cells needs to be further clarified. Previous studies have found that abnormal levels of lncRNAs are partly attributable to transcriptional activation regulated by acetylation [35–38]. In this study, the promoter region of DANCR showed that H3K27ac was obviously enriched in this region by bioinformatics analysis. Moreover, the level of H3K27ac at the DANCR promoter was highly increased and further upregulated DANCR expression in CRC tissue and cell lines. Histone acetyltransferases can control the histone acetylation process. In this study, we further used C646, a histone acetyltransferase inhibitor, to investigate the H3K27ac modification involved in DANCR expression. One study showed that C646 reduces H3K27ac enrichment at the DANCR promoter and causes downregulation of DANCR. These results indicate that the reason for DANCR upregulation is enrichment of H3K27ac at the DANCR promoter region. The limitation of this study was that other factors may function in the DANCR promoter region, such as methylation of H3K27 or lysine acetyltransferase 6A. We will further investigate to the association in CRC and publish the association in colorectal cancer patients in the future.

In summary, our study demonstrates that DANCR is activated by H3K27 acetylation and acts as an essential oncogene that promotes CRC proliferation. Mechanistically, DANCR functions as a miR-508-5p sponge to positively regulate ATF1 in CRC. This study reveals that DANCR is a potential therapeutic target in CRC.

### Supplementary Information

Supplementary file 1 (DOCX 17 KB)

## Data Availability

All the datasets generated and analyzed in the present study are included in this published article.

## Abbreviations

CRC: Colorectal cancer
lncRNA: Long noncoding RNA
miRNAs: MicroRNAs
ceRNAs: Competing endogenous RNAs
qPCR: Real-time quantitative polymerase chain reaction
siRNA: Short interfering RNA
