# Supplementary material for: Activation of lncRNA DANCR by H3K27 acetylation regulates proliferation of colorectal cancer cells
Source: Discov Oncol. 2024 Jun 28;15:249. doi: 10.1007/s12672-024-01124-8 (PMC11213841; doi:10.1007/s12672-024-01124-8)
Supplement: Supplementary file 1 — (DOCX 17 KB) [file 12672_2024_1124_MOESM1_ESM.docx]

1.Table S1. All mRNA primers sequences used in this study.

| Name | Sequence |
| --- | --- |
| NC/ NC mimic-F | AAUUCGUAGCUUGCAUGCAAGC |
| NC/NC mimic-R | CAGUACUUUGUGUAGUACAA |
| miR-508-5p mimic-F | TACTCCAGAGGGCGTCACTCATG |
| miR-508-5p mimic-R | AGTGCAGGGTCCGAGGTATT |
| NC inhibitor | CAGUACUUUUGUGUAGUACAA |
| ATF1-F | TCCTCACAGAAAGCCCACG |
| ATF1-R | CAGCAGCAGAAACTCCAGAAT |
| U6-F | CTC GCT TCG GCA GCA CA |
| U6-R | AAC GCT TCA CGA ATT TGC GT |
| GAPDH-F | CAT CCA TGA CAA CTT TGG TAT CGT |
| GAPDH-R | CCA TCA CGC CAC AGT TTC C |
| cyclin D1-F | GGCGGAGGAGAACAAACA |
| cyclin D1-R | ATGCAGGGCGGATTGGAAA |
| CDK4-F | TGAAATTGGTGTCGGTGCCT |
| CDK4-R | CAGTCGCCTCAGTAAAGCCA |
| Si-DANCR-F | CAAACUAACCCUACAGGUA |
| Si-DANCR-R | UACCUGCACGAAUUGAACG |
| Si-DANCR-F | GUCCUACAAGUACAGCUAU |
| Si-DANCR-R | CAAUGUUAACCUGAGGCUC |
